# Pure Ultrasound with Two-Step Dilation vs. Combined Ultrasound-Fluoroscopy in Percutaneous Nephrolithotomy: Is Fluoroscopy Still Needed?

**DOI:** 10.5152/tud.2026.25096

**Published:** 2026-03-03

**Authors:** Enrique Pulido-Contreras, Miguel Angel Garcia-Padilla, Carlos Rios-Melgarejo, Javier Medrano-Sanchez, Maria Guadalupe Leon-Verdin, Roger L. Sur

**Affiliations:** 1 Urology Department, Unidad Médica de Alta Especialidad No. 1 Bajío, Instituto Mexicano del Seguro Social, León, México; 2 Health Education Division, Unidad Médica de Alta Especialidad No. 1 Bajío, Instituto Mexicano del Seguro Social, León, México; 3 Research and Technological Development Department, Instituto de Salud Publica del Estado de Guanajuato, Guanajuato, México; 4 Department of Urology, University of California San Diego, California, USA

**Keywords:** Dilatation, fluoroscopy, nephrolithiasis, percutaneous nephrolithotomy, ultrasonography interventional

## Abstract

**Objective:**

Fluoroscopy has been the most widely used guidance method for percutaneous nephrolithotomy (PCNL); however, radiation exposure is a significant concern. Recently, ultrasound has gained popularity, offering several advantages. The objective was to evaluate the safety and outcomes of the pure-ultrasound PCNL with a two-step tract dilation.

**Material and Methods:**

The authors retrospectively analyzed the data of patients from March 2019 to October 2023. In all patients, percutaneous renal access was ultrasound guided, and they were divided into 2 groups: Group 1, all steps of PCNL were performed under ultrasound guidance, and Group 2, tract dilation, confirmation of stone clearance, and exit strategy were done with fluoroscopy.

**Results:**

The authors included 100 patients, 60 in Group 1 and 40 in Group 2, with a mean body mass index of 30.8 kg/m^2^. Mean stone burden was 4,162 mm^3^, 53% were complex stones and 59% of the cases were without hydronephrosis in the targeted calyx. Fluoroscopy was absent in Group 1 and 20 (11.3-31.5) seconds in Group 2 (*P* < .001). Complications were present in 13% of the cases. Group 1 patients had a lower complication rate than those in Group 2 (10% and 17%, respectively, *P* = .27). The authors had a global stone-free rate of 79%, Group 1: 78.3% and Group 2: 80% (*P* = 1.0). Multivariate analysis revealed that the stone-free rate is associated with the total operative time (*P* = .002) and the number of tracts (*P* = .031).

**Conclusion:**

Pure ultrasound PCNL is a safe and effective technique, offering a similar stone-free rate compared to fluoroscopy and providing an alternative method to avoid radiation exposure during PCNL.

**Main Points**

- Pure ultrasound guided PCNL with a two-step dilation technique achieves comparable stone-free and complication rates to fluoroscopy guided dilation PCNL.
- Fluoroscopy can be completely avoided during PCNL without compromising operative efficiency or perioperative outcomes.
- Stone-free status is mainly influenced by operative factors rather than the imaging modality used.

## Introduction

Percutaneous nephrolithotomy (PCNL) is the treatment of choice for large and complex stones.^1^ Fluoroscopy has been the most widely used imaging guidance method worldwide; however, radiation exposure is a significant concern, and multiple studies have demonstrated its negative effects on patients, the surgical team, and surgeons when exposed repeatedly, as radiation is cumulative. This chronic exposure to radiation carries a risk of developing cancer.^2^^,^^3^

To avoid radiation exposure, ultrasound has been proven to be a safe and reliable alternative imaging method to guide all steps when performing a PCNL. In China, ultrasound-guided PCNL (US_PCNL) was developed in 2004, completely avoiding the use of radiation.^4^ Subsequently, other Asian centers have developed additional ultrasound-guided techniques, and their use has gradually spread to other parts of the world.

Additionally, the use of US_PCNL can increase the number of hospitals where PCNL is performed, especially in hospitals where fluoroscopy is not available and ultrasound access is more feasible. This results in a cost reduction of approximately 30%.^5^

As previously reported, the authors successfully implemented ultrasound in their hospital in March 2019.^6^ Initially, the authors used it only for the puncture. Eventually, as the authors gained experience, in July 2021, the authors started using the ultrasound for all steps of the procedure. The objective of this study is to evaluate the safety and effectiveness of ultrasound-guided PCNL using a two-step dilation technique, compared to a mixed guided approach in which the puncture is performed under ultrasound, while dilation, residual stone search, and exit strategy are guided by fluoroscopy.

## Material and Methods

### Study Design and Patient Selection

**Figure 1.**
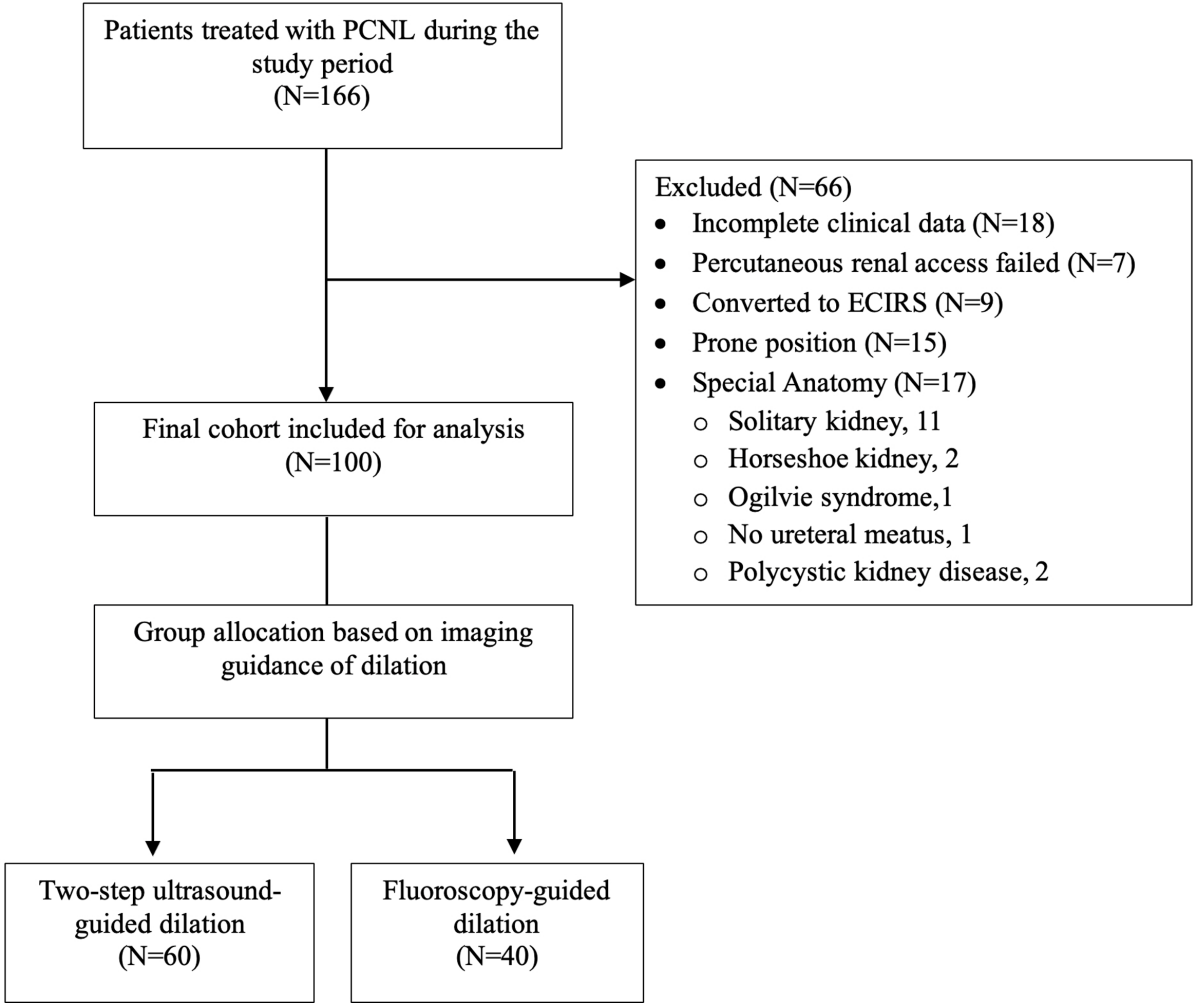
Flow diagram for retrospective study.

The authors conducted a retrospective cohort study of all patients who underwent PCNL at the institution from March 2019 to October 2023 with authorization from the hospital’s ethics and investigation committee (R-2024-1001-032). The inclusion criteria were patients aged 18 years or older with kidney stones. The authors excluded patients in whom ultrasound-guided percutaneous renal access failed, patients who underwent ECIRS, patients with renal abnormalities, patients in whom surgery was performed in prone position, and those in whom dilation was performed using balloon techniques or under endovision guidance. Patients were allocated to either Group 1 (two-step ultrasound-guided dilation) or Group 2 (fluoroscopy-guided dilation) based on the technique used. A flow diagram for group assignment is shown in Figure 1. Group allocation was primarily chronological, as the authors transitioned from the combined guided technique to the pure ultrasound-guided approach as the experience increased, with all cases after July 2021 performed using the totally ultrasound-guided technique.

All patients underwent preoperative computed tomography (CT) scans to determine stone characteristics. The skin-to-stone distance was calculated by measuring 3 distances from the center of the stone to the skin at 0º, 45º, and 90º, and the stone burden was calculated with the formula: “π × length × width × depth × 0.167.”^7^

### Surgical Procedure

All patients were placed in a supine position, allowing anterograde and retrograde access to the urinary tract. In addition to facilitating patient positioning, it allows the procedure to be performed under regional anesthesia. This began with the placement of a 5-Fr occlusion balloon for irrigation and generation of artificial hydronephrosis; the authors made sure the catheter is in the correct position using 2 maneuvers (seeing the catheter on the renal pelvis in the ultrasound or seeing the entrance of water to the caliceal system while they irrigate with solution).

In both groups, percutaneous renal access was ultrasound guided. The authors used a convex 3.5 MHz transducer (BK Medical Flex Focus 400, Denmark) and a configuration of the ultrasound machine (frequency, depth, focus, gain, and dynamic range) for adequate image quality. The authors performed the puncture using an 18G echogenic needle (Echotip Cook) with a free hand longitudinal technique, without the use of a needle guide, with the previously described technique.^8^

For the ultrasound-guided tract dilation with two steps, the authors describe 10 safety steps to increase the success of the procedure:

1. Successful puncture could be confirmed with urine flow.

2. Use an extra-stiff Amplatz guidewire 0.035″ (Cook Medical); it is easily visible on ultrasound.

Step 1 of dilation

3. Measure the distance from the skin to the collecting system using ultrasound.

4. Observe as the fascial dilators advance (10-12-14 Fr). They obscure the guidewire as they enter the depths of the calyceal system. Additionally, fascial dilators are marked in centimeters.

5. Creation of a mini tract with a 14-Fr peel-away sheath that will allow an anterograde nephroscopy using a 7.5-Fr rigid ureteroscope, ensuring we are inside the caliceal system. If the sheath is outside the calyceal system, advance the rigid ureteroscope over the guidewire to reach the calyceal system, then advance the 14-Fr peel-away sheath using continuous rotation over the ureteroscope. Incorrect guidewire positioning results in tract loss and necessitates repeating the puncture and dilation.

6. Using the ureteroscope, place two guidewires into the ureter: one for definitive dilation and a second as a safety wire.

7. Place the 14-Fr sheath at the entrance of the caliceal system to measure the distance to the skin (tract length); afterward, take out the 14-Fr sheath, fix the safety wire, and prepare the other wire for definitive dilation.

Step 2 of dilation

**Figure 2.**
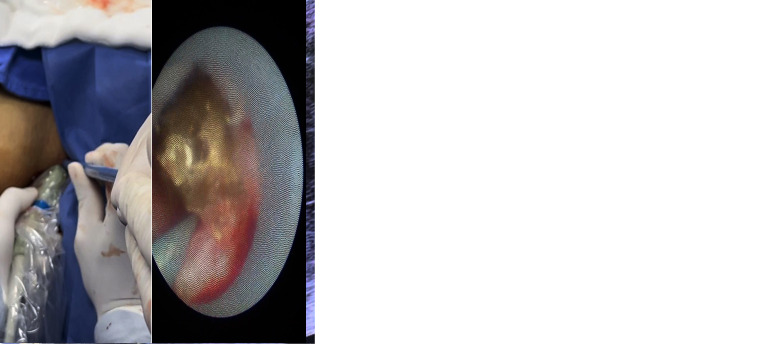
The two-step tract dilation technique: A) The advancement of fascial dilator (10-12-14 Fr) hides the guidewire and controls the depth of dilation. B) Antegrade nephroscopy with ureteroscope to check renal cavities and allocate 2 guides to the ureter, 1 final dilation and the other for security guidewire. C) The step-2 of dilation with amplatz 26 Fr.

8. Introduce the Alken antenna over the definitive dilation wire, and its tip is easily observed in the ultrasound as it passes the renal parenchyma and enters the calyceal system.

9. Use a 26-Fr Amplatz dilator for the definitive dilation, controlling its advancement using the ultrasound (as it goes inside, it generates an acoustic shadow) and place it 1 cm extra to the measure previously taken using the 14-Fr sheath, due to the mobility the kidney can have, reducing the possibility of a short dilation (Figure 2).

10. Place the Amplatz sheath up to the first mark on the dilator, which corresponds to its tip. This technique was clearly described in the previously published video.^9^

In the case of a short dilation, the authors advance the nephroscope between 2 guidewires. Once inside the calyceal system, they advance the Amplatz sheath, turning it around over the nephroscope. An alternative to this is the reported use of biprong forceps to advance the nephroscope.^10^

For fluoroscopy-guided dilation, the authors first advanced the guidewire, followed by a 10 Fr fascial dilator. Next, the Alken antenna is advanced into the renal cavities. Finally, a 26 Fr Amplatz dilator is inserted using the one-shot technique, all under fluoroscopic guidance.

To determine the stone-free rate, the authors order a low-dose CT scan a month after the surgery. Fragments ≤ 2mm are considered stone free. Complications were registered according to the Clavien–Dindo classification.

### Statistical Analysis

Baseline characteristics and outcomes were checked for normality using the Kolmogorov–Smirnov normality test with Lilliefors’ significance correction. Media and standard deviation were estimated for normal continuous variables. Median and interquartile range were calculated for non-normal continuous variables. Frequencies and proportions for categorical variables were assessed. Either 2-sided Student’s *T* or Mann–Whitney *U*-test was performed for continuous variables depending on normality. Fisher–Freeman–Halton test was computed for categorical variables. Multivariable logistic regression was used to determine the association, adjusting for the type of dilation. Data were analyzed using SPSS 27 with statistical significance set at *P* < .05.

## Results

### Patient Demographics and Stone Characteristics

The authors included 100 patients in the study, with 60 patients in group 1 (pure ultrasound guided) and 40 in group 2 (combined ultrasound fluoroscopy guided). The mean age was 49.6 ± 13 years, with most patients being female, comprising 54% of the population. Body mass index was 31.4 (26.9-34.8) and 28.7 (25.7-33.1) (*P* = .100) for each group, respectively. Regarding the stone characteristics, the stone burden was 4 726.3 (1 996.2-7 746.9) mm^3^ in Group 1 and 3 174.1 (1 621.2-8 755.4) mm^3^ in Group 2 (*P* = .412), with an average stone density of 1 077.5 ± 328.5 Hounsfield Units (HU). The authors observed some meaningful differences when comparing the proportions of the 2 groups. The Guy stone score was significantly different with a *P* = .045, indicating more complex stones (defined as Guy’s Stone Score 3-4) in Group 1. However, cases were arranged in chronological order to highlight the learning curve associated with the progressive adoption of the pure ultrasound-guided technique. The skin-to-stone distance was 9.97 ± 2.4 cm and 9.94 ± 2.2 cm (*P* = .94), respectively (Table 1).

### Operative Outcomes

Puncture time was longer in Group 1, with 45 (20-117.5) seconds in Group 1 and 25 (13.3-89) seconds in Group 2 (*P* = .022). Fluoroscopy time was 0 seconds in Group 1 and 20 (11.3-31.5) seconds in Group 2 (*P* < .001).

The puncture approach significantly differed between groups (*P* < .001). Group 1 patients more frequently received middle calyx puncture (43.3% vs. 15.0%), while lower calyx access was more common in group 2 (60% vs. 31.7%). Multiple tracts were created in a similar proportion of cases in both groups (15% vs. 20%, *P* > .007). Tract loss occurred in 5% of Group 1 and 0% of Group 2 (*P* = .273).

Total operative time did not differ significantly between groups, with a mean of 127.2 ± 48.7 minutes in Group 1 and 114.8 ± 49.5 minutes in Group 2 (*P* = .22). Hospital stay was (1-1 days) in both groups (*P* < .56). Most patients were managed tubeless, leaving the strings of the ureteral JJ stent exteriorized outside the percutaneous tract, a technique previously described and considered safe when removed within the first 8 postoperative days.^11^ When a nephrostomy tube was indicated for bleeding, patients were discharged with the tube, which was removed in the office 4 days postoperatively.

### Complications and Stone-Free Rates

Overall complications were present in 13% of the cases, with a lower rate in Group 1 (10%) compared to Group 2 (17.5%), though this difference was not statistically significant (*P* = .39). In Group 1, the authors had 1 patient with a grade 1 complication (fever), 3 patients with a grade 2 complication (bleeding that required blood transfusion), and 1 patient with a grade 3 complication (1 required a ureteroscopy for a ureteral stone). One patient had a grade 4 issue (sepsis that required admission to the ICU). In Group 2, 3 patients had a grade 1 complication (fever), and 4 patients had a grade 2 complication (bleeding that required blood transfusion). There was no difference in the descent of hemoglobin levels, with values of 1.7 ± 1.7 and 1.5 ± 1.3 g/dL, respectively (*P* = .76) (Table 2).

The stone-free rate was virtually identical between groups: 78.3% in group 1 vs. 80.0% in group 2 (*P* = .89). Residual stone burden was also similar: 268.6 (174.4-1038.3) mm^3^ in group 1 and 244.5 (171.87-524.7) mm^3^ in group 2 (*P* = .97).

The multivariable analysis revealed that complications were primarily associated with positive preoperative urine culture (OR 6.923, 95% CI 1.846-25.966, *P* < .004), with no significant associations between complications and two-step guided dilation, tract number, stone burden, and operative time (Table 3) and also revealed that the stone-free rate was associated with the short dilation (OR 0.065, 95% CI 0.011-0.386, *P* = 0.003), total operative time (OR 1.031, 95% CI 1.011-1.052, *P* = .002), and number of tracts (OR 0.042, 95% CI 0.002-0.750, *P* = .031) (Table 4).

## Discussion

The first report of ultrasound-guided access was published in 1974 by Pedersen for nephrostomy tube placement in an obstructed kidney.^12^ Later, in 1999, Mahesh Desai reported a series of pediatric patients in which percutaneous access guidance was made using ultrasound, and dilation was guided with fluoroscopy.^13^ In 2004, Li Jianxing described the two-step dilation technique, achieving totally ultrasound-guided PCNL and reporting its safety and effectiveness.^4^

Fluoroscopy continues to be the most commonly used imaging technique for PCNL guidance. However, ultrasound guidance is gaining popularity, particularly for the initial puncture, though increasingly for tract dilation as well. Both puncture accuracy and proper dilation are critical steps for successful PCNL outcomes.^13^^,^^14^ Given these trends and the known stochastic and deterministic risks of fluoroscopy, the data demonstrate the safety and efficacy of ultrasound guidance.

The effectiveness and results of the ultrasound-guided PCNL are similar to those reported with fluoroscopy^15^^,^^16^ ^,^^17^ while offering several distinct advantages: Beyond eliminating radiation exposure^18^ real-time visualization of the kidney and surrounding organs, including the colon, liver, spleen, and pleura.^19^ It also enables easier differentiation between posterior and anterior calyces compared to fluoroscopy.^20^ Additionally, Doppler ultrasound significantly reduces bleeding risk by identifying anomalous vascular patterns (including variations of the arcuate artery, ectopic arteries in the fornix, and segmentary artery variations) that are present in up to 35% of cases.^21^ Ultrasound guidance has proven particularly valuable in patients with complex spinal deformities^22^ and in the pediatric population^22^^,^^23^ Most importantly, no differences between the stone-free rate, complications, or hemoglobin descent between the ultrasound-guided dilation and the fluoroscopy-guided dilation were found.

In the study, it was demonstrated that tract dilation without fluoroscopy is feasible and safe. Even though tract dilation using ultrasound may seem challenging, it is feasible, safe, and straightforward.^24^^,^^25^ Currently, there are 3 main methods for tract dilation under ultrasound guidance:

1. Balloon dilation: As described by Chi et al^26^ the balloon can be monitored as it enters the calyceal system, with its location clearly visible when inflated.^25^ Pakmanesh et al compared Amplatz vs. balloon dilation under ultrasound guidance and found that Amplatz dilation increases the risk of short dilation. However, this risk was substantially lower with middle calyx access (18% vs. 61% with inferior calyx access), attributed to less kidney mobility.^10^ This finding supports the preference for middle calyx access, which offers reduced mobility and good nephroscope access to both inferior and superior calyces. One limitation of the balloon is that it may not be effective in patients who have undergone previous kidney surgery.^25^ Another limitation in the healthcare setting is the cost, which means it is not widely available.

2. Direct vision dilation (Endovision): This technique involves advancing a flexible ureteroscope to the target calyx for puncture and dilation control. However, it has limitations, including increased equipment requirements, higher costs, and reduced effectiveness in cases with high stone burden, stones impacted at the ureteropelvic junction, or difficulty advancing a flexible ureteroscope.^27^ Although all the patients underwent surgery in the supine position, which facilitates this approach under direct vision, the authors opted to perform the two-step dilation technique due to the limitations mentioned above and the inability to always have a flexible ureteroscope available.

3. Two-step dilation: This technique, initially described by Li Jianxing^4^ and subsequently modified by Zhou et al^25^ is our preferred approach. Zhou’s modification involves step 1: creating a 10 Fr mini-tract for antegrade nephroscopy using a 6-Fr ureteroscope to secure a wire into the ureter, followed by sequential dilation with fascial dilators (12, 14, 16 Fr) to place a 22-Fr working sheath in step 2. Our approach employs similar principles but with some technical variations as described in the methods. The reproduction of this technique in other countries has gained popularity, as it avoids radiation and reduces costs without compromising safety and clinical outcomes.^6^^28^ Alternatively, other Asian groups have described the feasibility and safety of dilation with Alken dilators using pure ultrasound guidance.^29^ Although the two-step dilation technique may seem more time-consuming, it is worthwhile. The miniaturized tract initially verifies that they are inside the collecting system. This allows to reposition the guides in the ureter before proceeding with the final standard dilation: one guide for dilation and the other as a safety guide. This enables to safely avoid radiation exposure.

As an alternative, dilation can be completed if mini percutaneous nephrolithotomy is to be performed only with step 1. However, as previously mentioned, most of the cases were complex and involved large stones. Therefore, despite having the necessary equipment and understanding the current tendency toward miniaturization, the authors still mainly use standard PCNL to resolve most of the cases.

### Learning Curve and Patient Selection

Some urologists have concerns about guiding the dilation with ultrasound. The most important points are having adequate visualization of the kidney and the guidewire. Adequate measurement of the stone-to-skin distance is also important to avoid excessive dilation.

Despite concerns of performing an ultrasound-guided puncture in obese patients,^30^ the authors have previously described a reproducible, safe technique, and their findings suggest that stone-free rates are more dependent on the stone characteristics than BMI.^9^

For surgeons starting their experience with ultrasound-guided PCNL, the authors recommend selecting ideal cases. Ideal initial cases include patients with hydronephrosis (which facilitates visualization and puncture) and non-staghorn stones (which simplifies the procedure). The authors also recommend having the fluoroscope available if needed. In our experience, during the transition from the combined to the pure ultrasound-guided technique, fluoroscopy was required in 15 of the first 40 cases. These included 9 staghorn calculi without hydronephrosis, 4 single kidneys, and 2 horseshoe kidneys. This initial phase reflects the learning curve associated with adopting the ultrasound-only approach. As experience increased, the authors were able to manage these complex cases without fluoroscopy, and beyond this 40 case transition period, fluoroscopy was no longer necessary.

Difficulties may arise when performing ultrasound-guided dilation; the authors had 3 patients with tract loss, but none in the fluoroscopy-guided dilation. A key to successful dilation is remembering the direction of needle entry during puncturing so that all dilators can be advanced in the same direction. This helps prevent the guide from accidentally exiting the kidney and helps avoid bending or kinking the guidewire, which can happen in kidneys with a large stone burden. This would subsequently cause the dilation to fail. The authors have noticed that, when dilating, if the authors no longer see the hyperechoic guidewire at the level of the renal parenchyma, the guidewire has likely exited the kidney. This requires puncture again and restarting the dilation. And in case of a short dilation in step 2, the authors advance the nephroscope between the 2 guidewires to complete dilation under direct vision. This concept parallels bi-prong endoscopic tract dilation, which recent data from Pakmanesh et al^31^ show to be safe and not associated with increased complications or inferior outcomes, supporting the usefulness of endoscopic salvage techniques when initial dilation is incomplete.

Additionally, during the learning curve phase, balloon dilation or endovision techniques may be easier to master before transitioning to the two-step technique. Performing US_PCNL without radiation requires a learning curve similar to that reported with fluoroscopy at around 60 cases.^32^

Regarding the exit strategy, the authors prefer a tubeless approach with a JJ stent left with external threads, allowing office removal. The authors leave the threads through the tract because this causes fewer urinary symptoms and improves patient comfort.^11^ In complicated cases with significant bleeding, weplace a nephrostomy tube or a nephrostomy plus ureteral stent, as recommended by the EAU guidelines.^1^

Our study has multiple limitations, including being a retrospective study without a control group. Another limitation was the different approaches to the exit strategy. To validate the data obtained from the study, a clinical trial involving multiple urologists with experience using ultrasound is required.

## Conclusions

The authors present data demonstrating that pure ultrasound-guided percutaneous nephrolithotomy using a two-step dilation technique is safe and effective, achieving outcomes comparable to the combined ultrasound-fluoroscopy approach while avoiding radiation exposure. With increasing experience, even complex cases can be successfully performed without the need for fluoroscopy.

## Figures and Tables

**Table 1.** Preoperative Descriptive Data

	**Total**	**Group 1**	**Group 2**	***P***
Patients	100	60	40	
Age, years, MV ± SD	49.6 ± 13.0	49.1 ± 11.9	50.4 ± 14.7	.608
Gender, n(%) Male Female	46 (46.0) 54 (54.0)	27 (45.0) 33 (55.0)	19 (47.5) 21 (52.5)	.840*
Body mass index, kg/m^2^, MD	30.8 (26.4-33.8)	31.4 (26.9-34.8)	28.7 (25.7-33.1)	.100^‡^
Laterality, n(%) Right Left	62 (62.0) 38 (38.0)	35 (58.3) 25 (41.7)	27 (67.5) 13 (32.5)	.405*
Location,n(%) Pelvic Lower calix Medium calix Upper calix Multiple Staghorn stone Partial staghorn	9 (9.0) 17 (17.0) 4 (4.0) 3 (3.0) 34 (34.0) 9 (9.0) 24 (24.0)	5 (8.3) 7 (11.7) 4 (6.7) 3 (5.0) 17 (28.3) 7 (11.7) 17 (28.3)	9 (10.0) 10 (25.0) 0 (0) 0 (0) 17 (42.5) 2 (5.0) 7 (17.5)	.102*
Stone burden, mm^3^, MD	4162.1 (1791.4-8089.0)	4726.3 (1996.2-7746.9)	3174.1 (1621.2-8755.4)	.412^‡^
Stone density, HU, MV ± SD	1077.5 ± 328.5	1125.8 ± 334.7	1005.0 ± 308.9	.071
Skin-to-stone distance, cm, SD	9.96 ± 2.3	9.97 ± 2.4	9.94 ± 2.2	.944
Guy Stone,n (%) Guy Stone I Guy Stone II Guy Stone III Guy Stone IV	27 (27.0) 20 (20.0) 38 (38.0) 15 (15.0)	15 (25.0) 8 (13.3) 29 (48.3) 8 (13.3)	12 (30.0) 12 (30.0) 9 (22.5) 15 (17.5)	.041*
Degree, hydronephrosis, n(%) Normal Grade I Grade II Grade III	59 (59.0) 23 (23.0) 17 (17.0) 1 (1.0)	38 (63.3) 12 (20.0) 10 (16.7) 0 (0.0)	21 (52.5) 11 (27.5) 7 (17.5) 1 (2.5)	.455*
Preoperative hemoglobin, g/dL, SD	14.6 ± 2.1	14.5 ± 2.0	14.4 ± 2.2	.541
Urine culture preop, n(%) Negative Positive	53 (53.0) 47 (47.0)	29 (48.3) 31 (51.7)	24 (60.0) 16 (40.0)	.308*

Data are presented as mean (MV) and standard deviation (SD), median (MD), and interquartile range (IQR) or frequency or a percentage.

^‡^U de Mann–Whitney test: Asymptotic significance was reported.

*Exact bilateral significance for Fisher–Freeman–Halton test.

**Table 2.** Perioperative and Postoperative Data

	**Total**	**Group 1**	**Group 2**	***P***
Ultrasound puncture time, seg, MD	39.5 (16-107.5)	45 (20-117.5)	20 (13.3-89.0)	.022^‡^
Fluoroscopy time, seg, MD	0 (0-16.0)	0	20 (11.3-31.5)	<.001^‡^
Furosemide before puncture, n (%)	75 (75.0)	48 (80.0)	27 (67.5)	.167*
Irrigation by ureter for dilation, n (%)	88 (88.0)	54 (90.0)	34 (85.0)	.535*
Puncture approach: n (%) Lower calix Middle calix Upper calix Multiple tracts	43 (43.0) 32 (32.0) 8 (8) 17 (17.0)	19 (31.7) 26 (43.3) 6 (10.0) 9 (15.0)	24 (60.0) 6 (15.0) 2 (5.0) 8 (20.0)	.007*
Tract loss: n (%)	3 (3.0)	3 (5.0)	0 (0.0)	.273*
Total operative time, minutes, MV ± SD	122.2 ± 49.2	127.2 ± 48.7	114.8 ± 49.5	.218
Postoperative hemoglobin, g/dL, SD	13.0 ± 2.3	13.0 ± 2.3	12.9 ± 2.5	.764
Difference in hemoglobin drop, g/dL, MD (IQR)	1.5 (0.8-2.5)	1.5 (0.9-2.6)	1.5 (0.4-2.4)	.691
Complication Clavien–Dindo, n (%) Grade 1 Grade 2 Grade 3 Grade 4	4 (4.0) 7 (7.0) 1 (1.0) 1 (1.0)	1 (1.7) 3 (5.0) 1 (1.7) 1 (1.7)	3 (7.5) 4 (10.0) 0 0	.389*
Flexible nephroscopy, n (%) Flexible cystoscope anterograde	56 (56.0)	45 (75.0)	11 (27.5)	<.001*
Drainage type, n (%) Total tubeless Ureteral stent, threads by tract Ureteral stent, threads by urethra Ureteral stent without threads Nephrostomy tube Ureteral stent + nephrostomy tube	1 (1.0) 59 (59.0) 24 (24.0) 3 (3.0) 8 (8.0) 5 (5.0)	0 33 (55.0) 20 (33.3) 1 (1.7) 2 (3.3) 4 (6.7)	1 (2.5) 26 (65.0) 4 (10.0) 2 (5.0) 6 (15.0) 1 (2.5)	.009*
Days to remove drainage, MV ± SD	4.2 ± 1.7	4.4 ± 1.6	4.0 ± 1.9	.242
Stone free status, n (%)	79 (79.0)	47 (78.3)	32 (80.0)	1.00*
Volume of residual stone, mm^3^, MD (IQR)^†^	262.3 (174.4-631.7)	268.6 (174.4-1038.3)	244.5 (171.87-524.7)	.972^‡^
Hospital stay, days, MD (IQR)	1 (1-1)	1 (1-1)	1 (1-1)	.56^‡^
Type of anesthesia, n (%) Regional General	33 (33.0) 67 (67.0)	19 (31.7) 26 (68.3)	14 (35.0) 26 (65.0)	.829*
Urine culture postoperative, n (%)^a^ Negative Positive	79 (79.0) 21 (21.0)	46 (76.7) 14 (23.3)	33 (82.5) 7 (17.5)	.618*

^a^Urine culture was taken 1 month after the surgery.

Data are presented as mean (MV) and standard deviation (SD), median (MD) and interquartile range (IQR), or a percentage.

^‡^U de Mann–Whitney test: Asymptotic significance was reported.

*Exact bilateral significance for Fisher-Freeman-Halton test.

**Table 3.** Multivariable Analysis for Complications (n = 100)

**Variable**	**OR**	**95% CI**	***P***
Two-step ultrasound dilation (ref one-shot fluoroscopy)	0.98	0.244-16.058	.522
Tract number	0.481	0.080-2.887	.424
Positive preoperative urine culture	6.923	1.846-25.966	.004*
Stone burden (mm^3^)	1.000	1.000-1.000	.119
Operative time (minutes)	1.010	0.995-1.025	.209

*Statistically significant at <0.05

**Table 4.** Multivariable Analysis for Residual Stone (n = 100)

**Variable**	**OR**	**95% CI**	***P***
Stone localization Pelvis Lower calyx Middle and superior calyx Multiple calyx Staghorn complete or incomplete	1.146 1.297 7.857 14.723 0.994	0.077-17.105 0.092-18.202 0.210-293.597 0.544-398.283 0.244-4.052	.921 .847 .265 .110 .994
Puncture localization Lower calyx Middle calyx Upper calyx	0.029 0.090 0.065	0.001-1.028 0.002-3.556 0.001-3.967	.052 .199 .193
Short dilation	0.065	0.011-0.386	.003*
Total operative time	1.031	1.011-1.052	.002*
Tract number	0.042	0.002-0.750	.031*
Stone burden (mm^3^)	1.000	1.000-1.000	.122

*Statistically significant at <0.05

## Data Availability

The data that support the findings of this study are available on request from the corresponding author.
